# Analysis of a macrophage carbamylated proteome reveals a function in post-translational modification crosstalk

**DOI:** 10.21203/rs.3.rs-3044777/v1

**Published:** 2023-06-16

**Authors:** Youngki You, Chia-Feng Tsai, Rishi Patel, Soumyadeep Sarkar, Geremy Clair, Mowei Zhou, Tao Liu, Thomas O. Metz, Chittaranjan Das, Ernesto S. Nakayasu

**Affiliations:** Pacific Northwest National Laboratory; Pacific Northwest National Laboratory; Purdue University; Pacific Northwest National Laboratory; Pacific Northwest National Laboratory; Pacific Northwest National Laboratory; Pacific Northwest National Laboratory; Pacific Northwest National Laboratory; Purdue University; Pacific Northwest National Laboratory

## Abstract

**Background.:**

Lysine carbamylation is a biomarker of rheumatoid arthritis and kidney diseases. However, its cellular function is understudied due to the lack of tools for systematic analysis of this post-translational modification (PTM).

**Methods.:**

We adapted a method to analyze carbamylated peptides by co-affinity purification with acetylated peptides based on the cross-reactivity of anti-acetyllysine antibodies. We integrated this method into a mass spectrometry-based multi-PTM pipeline to simultaneously analyze carbamylated and acetylated peptides in addition to phosphopeptides were enriched by sequential immobilized-metal affinity chromatography.

**Results.:**

By testing the pipeline with RAW 264.7 macrophages treated with bacterial lipopolysaccharide, 7,299, 8,923 and 47,637 acetylated, carbamylated, and phosphorylated peptides were identified, respectively. Our analysis showed that carbamylation occurs on proteins from a variety of functions on sites with similar as well as distinct motifs compared to acetylation. To investigate possible PTM crosstalk, we integrated the carbamylation data with acetylation and phosphorylation data, leading to the identification 1,183 proteins that were modified by all 3 PTMs. Among these proteins, 54 had all 3 PTMs regulated by lipopolysaccharide and were enriched in immune signaling pathways, and in particular, the ubiquitin-proteasome pathway. We found that carbamylation of linear diubiquitin blocks the activity of the anti-inflammatory deubiquitinase OTULIN.

**Conclusions:**

Overall, our data show that anti-acetyllysine antibodies can be used for effective enrichment of carbamylated peptides. Moreover, carbamylation may play a role in PTM crosstalk with acetylation and phosphorylation, and that it is involved in regulating ubiquitination *in vitro*.

## Background

Carbamylation (also known as carbamoylation) is a modification of lysine residue side chains, generating Nε’-carbamyl-lysine or homocitrulline [1]. To date, all known lysine carbamylation of protein and peptides are products of non-enzymatic reactions induced by cyanate and isocyanic acid, formed from urea and thiocyanate, respectively, or by carbamoyl phosphate, an intermediate metabolite of arginine metabolism and nucleotide biosynthesis [1, 2]. Cyanate can be formed in the body by uremia in kidney disease, while isocyanic acid is a product of the pro-inflammatory enzyme myeloperoxidase whose expression is elevated in infectious or autoimmune diseases, such as rheumatoid arthritis. As a result, carbamylation is considered a biomarker for these diseases [3–6]. Despite carbamylation links to various diseases, its roles in pathogenesis and physiology are understudied, mainly due to the lack of tools available for systematic analysis of its function.

Proteomics has been an important tool for studying a variety of protein post-translational modifications (PTMs). However, carbamylation represents a major hurdle for the proteomics community. Carbamylation can be generated as an artifact when denaturing proteins with urea [7], a crucial step for efficient proteolysis during proteomics sample preparation. In addition, carbamylation is a major contaminant and confounding factor of lysine acetylome analysis. Lysine acetylation (+ 42.0103 Da) and carbamylation (+ 43.00543 Da) have similar mass; there is particular overlap in mass when comparing carbamylation to the ^13^C isotope of acetylation (+ 43.0137 Da), a difference of only 8 mDa. Thus, mis-identifications can occur depending on the database searching tool [8]. Another issue is that peptides containing lysine carbamylation can be co-purified with those containing acetylation when using anti-acetyllysine antibodies due to their structural similarities [9].

In this study, we sought to investigate the roles of carbamylation in the RAW 264.7 macrophage cell line by performing a global analysis of this PTM in response to an inflammatory stimulus with bacterial lipopolysaccharide. We took advantage of the co-purification of acetylation and carbamylation to simultaneously analyze both PTMs and performed isotope correction and recalibration to accurately distinguish between the two. We also integrated the data with phosphorylation through a sequential phosphopeptide enrichment of the same sample and further analyzed the data to investigate possible PTM crosstalk and pathways that they might affect. Our data show that carbamylation can be effectively enriched with anti-acetyllysine antibodies. In addition, we showed some characteristics of protein carbamylation and identified a potential role in crosstalk with other PTMs.

## Methods

### Cell culture and treatments

RAW 264.7 cells were cultivated in DMEM medium containing 10% fetal bovine serum and penicillin/streptomycin at 5% CO_2_ atmosphere at 37 °C. Cells were treated with 100 ng/mL *Salmonella* lipopolysaccharide (Invitrogen, cat. No. 00-4976-93) for 24 h at 5% CO_2_ atmosphere at 37 °C. Cells were washed twice with cold PBS (4 °C), scraped, and harvested into centrifuge tubes. Cells were centrifuged for 5 min at 500 g, the supernatant was discarded, and the pellet was stored at −80 °C for multi-omics analysis.

### Protein digestion, labeling and peptide enrichment

Cell pellets were lysed in 50 mM triethylammonium bicarbonate buffer containing 8 M urea or 12 mM sodium deoxycholate (SDC) at 4 °C for 15 min followed by 95 °C for 5 min. Cell lysates were reduced with 10 mM dithiothreitol at room temperature for 30 min, and cysteine residues were alkylated with 50 mM iodoacetamide at room temperature for 30 min, protected from the light. The reaction was diluted 5-fold with 50 mM triethylammonium bicarbonate buffer and digested with 1:25 trypsin:protein ratio and 1:50 endoproteinase Lys-C/protein ratio overnight at room temperature. Enzyme reactions were stopped by adding trifluoroacetic acid (0.5% final concentration). Peptides were desalted by solid phase extraction using C18 cartridges (Phenomenex) and dried in a vacuum centrifuge. An optimized ratio of TMT to peptide amount of 1:1 (w/w), recently reported by Zecha et al. [10] was used, and samples were fractionated by high pH reverse phase chromatography and concatenated into 12 fractions. Carbamylated and acetylated peptides were enriched with anti-acetyllysine antibodies using PTMScan^®^ Acetyl-Lysine Motif Immunoaffinity Beads (Cell Signaling), following manufacturer recommendations. For analysis of all three PTMs, the samples were first subjected to phosphopeptide enrichment using a recently developed tip-based immobilized metal affinity chromatography (IMAC) method [11], followed co-enrichment of carbamylated and acetylated peptides from the IMAC flow-through using the procedure described above.

### Mass spectrometry and data analysis

Peptides dissolved in 2% acetonitrile and 0.1% trifluoroacetic acid were separated using a reversed-phase column (packed in-house into a 25-cm length of 360 μm o.d. × 75 μm i.d. fused silica picofrit New Objective capillary tubing using ReproSil-Pur 120 C18-AQ 1.9 μm stationary phase) connected to a nanoACQUITY UPLC system (Waters). The analytical column was heated to 50°C using an AgileSLEEVE column heater (Analytical Sales and Services). Peptides were separated through a linear gradient from 8–35% buffer B over 100 min at a flow rate of 200 nL/min. MS analysis was performed using an Orbitrap Fusion Lumos mass spectrometer (ThermoFisher Scientific). Orbitrap precursor spectra (AGC 4×10^5^) were collected from 350–1800 m/z for 110 min at a resolution of 60K along with data-dependent Orbitrap HCD MS/MS spectra (centroid) at a resolution of 50K (AGC 1×10^5^). For acetylation and carbamylation peptide analyses, max ion injection time was set at 125 ms. For phosphopeptide analysis, max ion injection time was set at 105 ms. The total duty cycle was 2 seconds. Precursor ions for MS/MS were isolated (quadrupole) at a width of 0.7 m/z and fragmented using a normalized collision energy of 30%. Peptide mode was selected for monoisotopic precursor scan and charge state screening was enabled to reject unassigned 1 + and > 7+-charged ions with a dynamic exclusion time of 45 seconds. Data were processed with MaxQuant software (v2.1.0.0) [8] by matching against the mouse reference proteome database from Uniprot Knowledge Base (downloaded on August 31, 2020). Searching parameters included protein N-terminal acetylation and oxidation of methionine as variable modifications, and carbamidomethylation of cysteine residues as fixed modification. Searching parameters included protein N-terminal acetylation and oxidation of methionine as variable modifications, and carbamidomethylation of cysteine residues as fixed modification. For phosphoproteome analysis, phosphorylation of serine, threonine and tyrosine residues was set as a variable modification. Acetylation and carbamylation of lysine residues were set as variable modifications. For the motif analysis (see below) data processed with MSFragger (v3.5) [12] using FragPipe (v18.0) with the same parameters than the MaxQuant analysis.

### Statistical and pathway analysis

Reporter ion intensity values were normalized by median centering before submitting to Student’s t-test. Functional-enrichment analysis was done with Database for Annotation, Visualization and Integrated Discovery (DAVID) [13], using the KEGG annotation. Connectivity between proteins was queried in the String database.[14] Additional pathway analysis for the proteins which showed carbamylation, acetylation, phosphorylation was conducted using Reactome [15].

### Motif analysis

The unique sequences of carbamylated or acetylated lysine residues at the center ± seven adjacent residues. The carbamylation and acetylation motifs were generated with 6,455 carbamylated sites and 5,278 acetylated sites using pLogo (v1.2.0) [16] and 636,113 mouse sequences in pLogo were used for the background sequences. We regenerated the additional motifs after fixing a specific residue that was placed at the ± 1 site of carbamylated or acetylated lysine residues and ranked within the top three in the first motif analysis.

### Structural analysis

Protein structures were downloaded from the Protein Data Bank (PDB) and analyzed with Discovery Studio Visualizer 4.5 program.

### Cloning, expression, and purification of recombinant proteins

OTULIN^FL^ and M1-linear diubiquitin was cloned into expression plasmids pCOLD-HisSUMO (Takara Bio) and pET-26b respectively. These plasmids were transformed into *E. coli* BL21 DE3 and plated against LB-agar containing 100 μg/mL ampicillin and 50 μg/mL kanamycin respectively. A single colony was inoculated into LB media containing the respective antibiotics and grown overnight at 37°C. Cells were grown with shaking at 37°C until an OD_600_ = 0.6–0.8 was reached. Protein expression was induced with 0.35 mM IPTG for 18 h at 18°C. Cells expressing OTULIN^FL^ were harvested at 7000 rpm for 10 min and were resuspended in pH 7.4 phosphate-buffered saline (PBS) with 400 mM KCl containing 0.5 mg/mL lysozyme and lysed using a French press. Lysate was clarified by ultracentrifugation for 1 h at 100,000 g at 4°C and applied to 5 mL of Ni-NTA agarose (Qiagen) resin pre-equilibrated with the respective lysis buffer. The resin is washed with 20 column volumes (CV) of 1X PBS 400 mM KCl, 20 CV of 1X PBS 400 mM KCl containing 25 mM imidazole, and finally eluted with 8 CV of 1X PBS 400 mM KCl containing 300 mM imidazole. The elution was concentrated, and buffer exchanged 1X PBS 1 mM DTT using an Amicon 10 kDa molecular weight cut-off concentrator (Millipore-Sigma). Cells expressing M1-linear diubiquitin were resuspended in 50 mM sodium acetate pH 4.5 and disrupted by French press as described earlier. Cell lysates were heated to 70–80°C for 15 min prior to ultracentrifugation as described above. The clarified supernatant was applied to a self-packed SP Sepharose Fast Flow resin (GE Healthcare) column and eluted with a gradient 1 M NaCl in 50mM sodium acetate buffer. Protein fractions had the purity determined by SDS-PAGE analysis, and were pooled, concentrated, and exchanged into 1X PBS. All protein purity and homogeneity described here is monitored by SDS-PAGE.

### M1-linear Diubiquitin Carbamylation

Carbamylation of M1-linear diubiquitin, purified as described earlier, was carried out by incubation of the dimer in 0.1 M potassium cyanate (AK Scientific) in 1X PBS pH 7.4 at 37°C for 24 h. The carbamylated M1-linear diubiquitin was buffer exchanged by size exclusion chromatography the next day into 1X PBS. Carbamylation was assessed by direct infusion on an Orbitrap Exploris 480 mass spectrometer (Thermo Fisher Scientific). The protein solution was desalted using Amicon Unltra 0.5 mL centrifugal filters (MWCO 3 kDa, Millipore). Then the diluted protein solution at ~ 0.1 μM in 50% acetonitrile 0.1% formic acid was infused at 3 μL/min with HESI source at sheath gas 5, auxiliary gas 7, spray voltage 3.2 kV, ion transfer tube at 320°C, and funnel RF level at 50%. Spectra were acquired at 240k resolution setting in positive mode, and deconvoluted by Xtract within FreeStyle v1.5 (Thermo Fisher Scientific).

### Deubiquitylating Assay

OTULIN activity towards the substrates unmodified and carbamylated M1-linear diubiquitin was carried out in 1X PBS 1mM DTT buffer. The reactions were started by adding equal volumes of enzyme (500 pM final concentration) and substrate (20 μM final concentration) solutions. The reaction was quenched at differing time points (t = 0, 1, 6, and 24 h) by the addition of 5X SDS-PAGE loading dye. The experiment was carried out in triplicate.

## Results

### Establishing a method for global lysine carbamylation analysis

**Figure 1:**
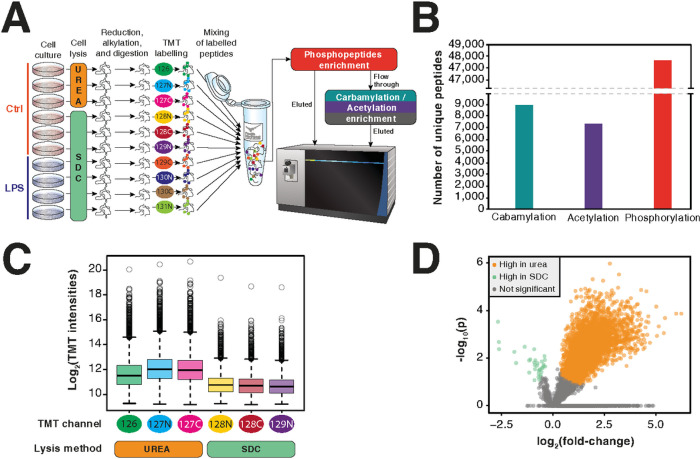
Quantitative multi-post-translational modification analysis of RAW 264.7 cells treated with bacterial lipopolysaccharide. (a) Workflow of the sample preparation procedure and proteomics analysis of phosphorylated, carbamylated and acetylated peptides. (b) Number of unique carbamylated, acetylated, and phosphorylated peptides. (c) Box plot of intensities of carbamylated peptides obtained from samples prepared with urea or sodium deoxycholate (SDC) denaturing. (d) Volcano plot of carbamylated peptides intensities from samples denatured with urea vs. SDC.

To establish a lysine carbamylation enrichment method, we tested the ability of anti-acetyllysine antibody to co-capture carbamylated peptides (Fig. 1A). We digested RAW 264.7 cell lysate replicates in buffer containing urea or SDC as denaturing agents. Urea causes carbamylation *in vitro* and therefore was used as a positive control for carbamylation, whereas SDC does not induce carbamylation and therefore, it was used to detect endogenous sites. After digestion, peptides were labeled with TMT and phosphopeptides were captured by IMAC. The unbound fraction from the IMAC had both acetylated and carbamylated peptides co-captured with anti-acetyllysine antibodies. All fractions were then analyzed by LC-MS/MS. To distinguish between carbamylation and acetylation, we used MaxQuant software, which automatically performs isotope correction of parent ions, recalibrates the mass spectrometry measurements, and performs searches with 4.5 ppm mass tolerance. This process reduces the chances of mismatching carbamylation and acetylation. A total of 63,859 modified peptides were identified, including 7,299, 8,923, and 47,637 acetylated, carbamylated, and phosphorylated peptides, respectively (Fig. 1B, Tab. S1–3). The quantitative analysis showed that the samples digested in buffer containing urea had carbamylated peptides with 2 logs (4-fold) higher average intensity of the reporter ions, confirming that they are in fact carbamylated (Fig. 1C–D). These results showed that anti-acetyllysine can efficiently enrich carbamylated peptides in addition to acetylated peptides. Furthermore, our pipeline provides in-depth coverage of multiple PTMs from the same samples.

### Pathways differentially carbamylated in RAW 264.7 cells treated with LPS

**Figure 2:**
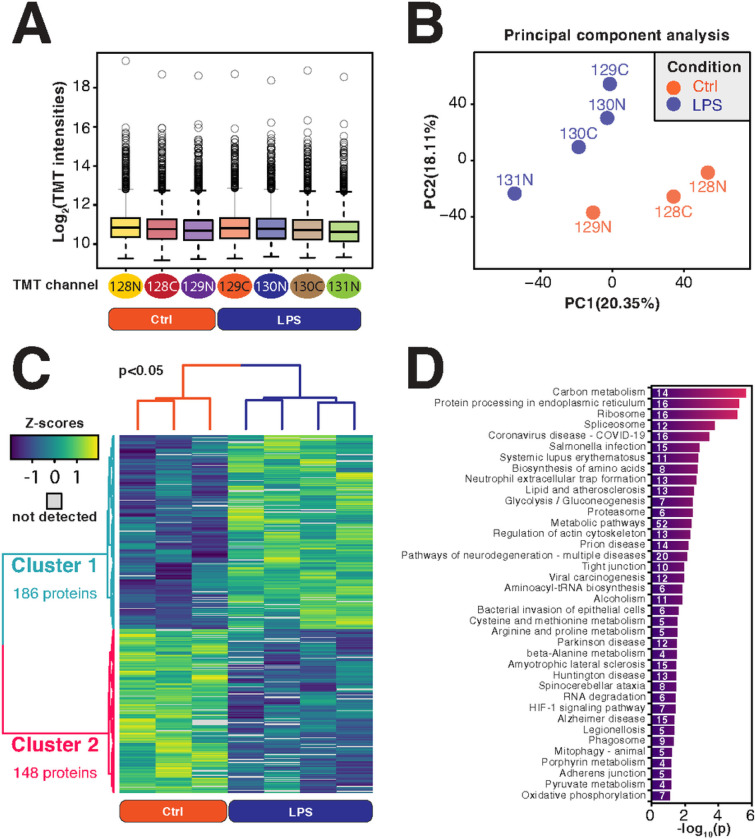
Carbamylated proteomics analysis of macrophages treated with bacterial lipopolysaccharide (LPS). (a) Box plot of carbamylated peptide intensities of LPS-treated vs untreated group. (b) Principal component analysis of carbamylated peptides from LPS-treated and control samples. (c) Heatmap of carbamylated peptides of LPS-treated and control samples. (d) A DAVID functional-enrichment analysis of proteins with carbamylation sites significantly regulated by the LPS treatment.

Out of the 8,468 quantifiable carbamylated peptides, 2,378 proteins were found in the samples digested with SDC, showing that carbamylation occurs endogenously in a large number of proteins in cells. To study possible functions of carbamylation in inflammation, we treated RAW 264.7 cells with bacterial lipopolysaccharide (LPS) and analyzed these in parallel with untreated controls (Fig. 2). The carbamylated peptides of both control and LPS treatment groups showed similar average intensity of TMT reporter ions (Fig. 2A). A principal component analysis showed that the carbamylated peptides of the LPS treatment group were clustered together and segregated from the carbamylated peptides of the control group (Fig. 2B). The quantitative analysis showed that 195 endogenously carbamylated peptides from 186 proteins were upregulated by the LPS treatment, while 165 endogenously carbamylated peptides from 148 proteins were downregulated (Fig. 2C). A functional-enrichment analysis showed an overrepresentation of differentially abundant carbamylation in proteins in 38 pathways (Fig. 2D). This included a variety of metabolic pathways (e.g., carbon, amino acid, and porphyrin metabolisms), protein synthesis and processing (e.g., ribosomes and protein processing in the endoplasmic reticulum), RNA synthesis, processing, and degradation (e.g., spliceosome, t-RNA biosynthesis, and RNA degradation), and signaling pathways (e.g., HIF-1 signaling pathway) (Fig. 2D). These results showed that carbamylated proteins from a variety of processes are regulated in the cells by LPS, ranging from cellular metabolism to protein synthesis and signaling pathways.

### Endogenous carbamylation motif analysis

**Figure 3:**
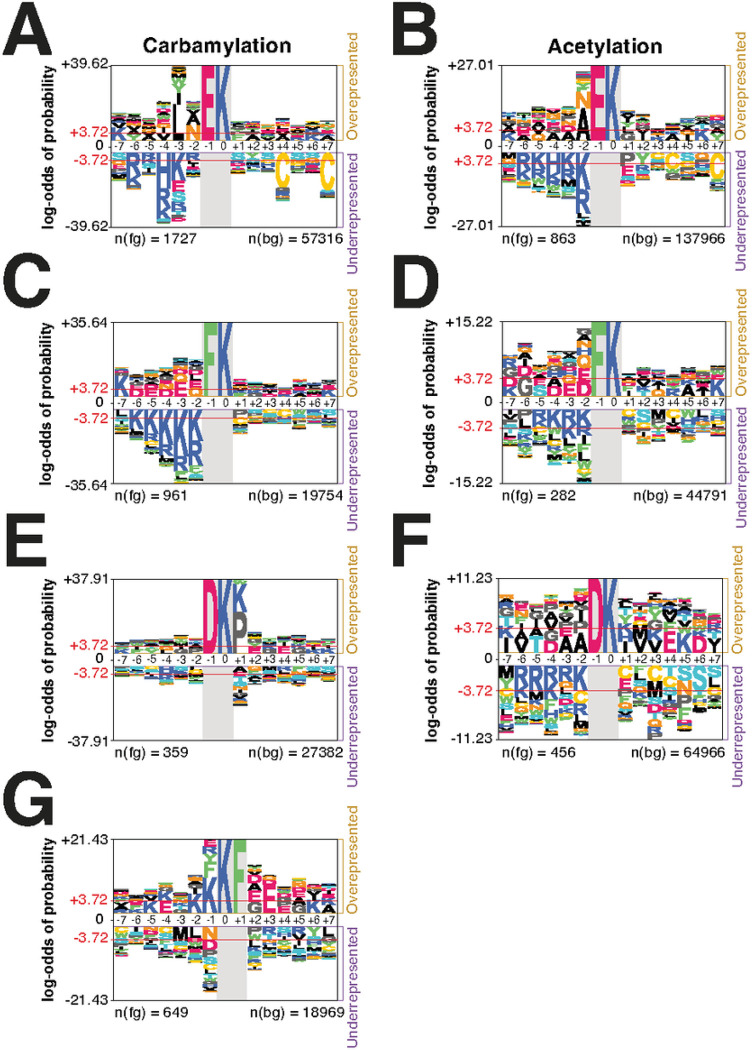
Motif analysis of carbamylation site. Sequence motif logo of carbamylated lysine (a) and acetylated lysine (b). Glutamic acid (c and d), phenylalanine (e and f), or aspartic acid (g and h) at −1 position from the carbamylated or acetylated lysine was fixed in the sequence logos. Phenylamine at +1 position from the carbamylated lysine was fixed in the sequence logo (i).

We performed a motif analysis to study carbamylation specificity and to compare against acetyllysine motifs (Fig. 3). Lysine carbamylation was significantly enriched with glutamic acid or phenylalanine at the − 1 position. In the case of glutamic acid, carbamylation occurred nearby hydrophobic residues (Fig. 3A). Acetyllysine was also significantly enriched with glutamic acid at the − 1 position, but an adjacent hydrophobic residue was not as evident (Fig. 3B). Negatively charged residues (aspartic and glutamic acids) were overrepresented nearby carbamylated lysine sites with phenylamine at the – 1 position (Fig. 3C). The same was observed for acetyllysine (Fig. 3D). Positively charged residues (arginine and lysine) were underrepresented near the modified lysine in both motifs with both glutamic acid and phenylalanine at the − 1 position. We also found carbamylation and acetylation motifs containing aspartic acid at the − 1 position. However, in the case of carbamyllysine, this motif was accompanied by an enrichment of proline or lysine at the + 1 position (Fig. 3E–F). In carbamylation, another motif was enriched with phenylalanine at the + 1 position, with adjacent negatively charged amino acids (Fig. 3G). These results show that carbamylation occurs preferentially at the lysine adjacent to negatively charged residues nearby hydrophobic ones.

### Integration of carbamylation with acetylation and phosphorylation

**Figure 4:**
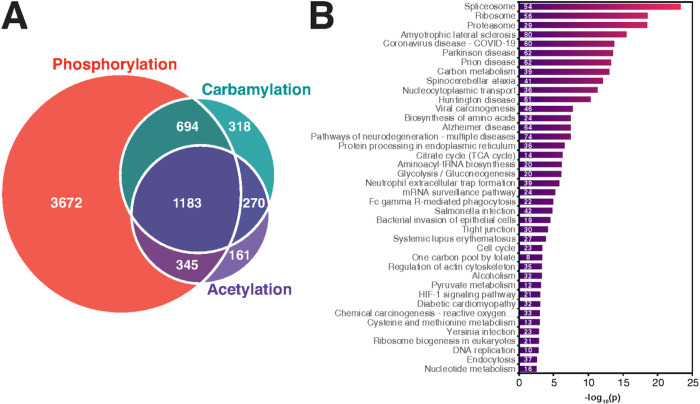
Number of proteins modified with carbamylation, acetylation, or phosphorylation and a functional enrichment of proteins commonly modified by all three post-translational modifications. (a) Venn diagram of carbamylated, acetylated, or phosphorylated proteins. (b) A functional-enrichment analysis of the 1183 proteins commonly modified with all three post-translational modifications.

**Figure 5:**
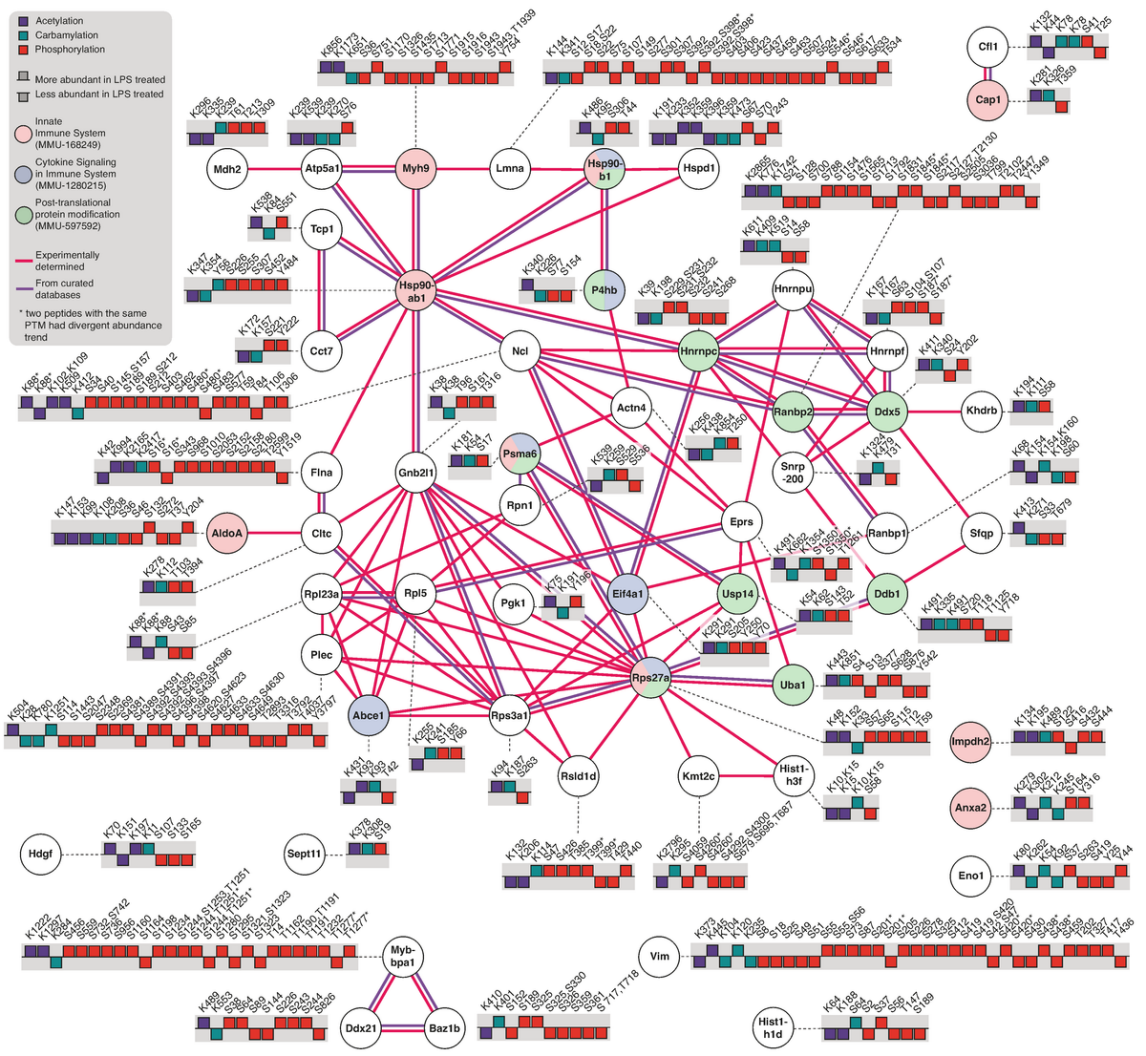
Protein-protein interaction network and functional enrichment analysis of proteins with acetylation, carbamylation and phosphorylation sites regulated by bacterial lipopolysaccharide. The network contains 54 common proteins which had altered levels of carbamylation, acetylation, and phosphorylation by the LPS treatment. The bar graph shows the direction of regulation (p < 0.05) of the three modifications after LPS treatment. The network was enriched in proteins of the innate immune system, cytokine signaling pathway in immune system, and protein post-translation modification pathway using Reactome and they are highlighted the color of pink, blue, green, respectively. The line colors represent if the interactions were experimental determined (pink) and curated in databases (purple).

We next investigated possible carbamylation crosstalk with acetylation and phosphorylation by searching for proteins that were commonly modified by these 3 PTMs. We found 1,183 proteins that were commonly modified by all 3 PTMs (Fig. 4A) and they were overrepresented in a variety of pathways such as metabolic pathways and protein degradation pathways (Fig. 4B). Among the 1,183 commonly modified proteins, 54 proteins had the levels of all 3 PTMs regulated by the LPS treatment. We queried the String database to investigate if these proteins were somehow involved in similar functions (Fig. 5). The analysis reviewed a high connectivity between the proteins, indicating that they interact or participate in the same pathways. Of these 54 proteins, 14 were signaling proteins of the immune system (p = 0.0035), and 6 were from the cytokine signaling (p = 0.0184), based on Reactome pathway analysis. There was also an enrichment of proteins related to PTMs involved in the ubiquitin-proteasome pathway, including proteasome subunit alpha type-6 (Psma6), ubiquitin carboxyl-terminal hydrolase 14 (Usp14), Ubiquitin-40S ribosomal protein S27a (Rps27a) (ubiquitin), ubiquitin-activating enzyme E1 (Uba1), and E3 SUMO protein ligase (RanBP2) (Fig. 5). These results suggest a regulation of immune signaling pathways by carbamylation, phosphorylation and acetylation via another PTM, i.e. ubiquitination.

### Effect of carbamylation on protein ubiquitination

**Figure 6:**
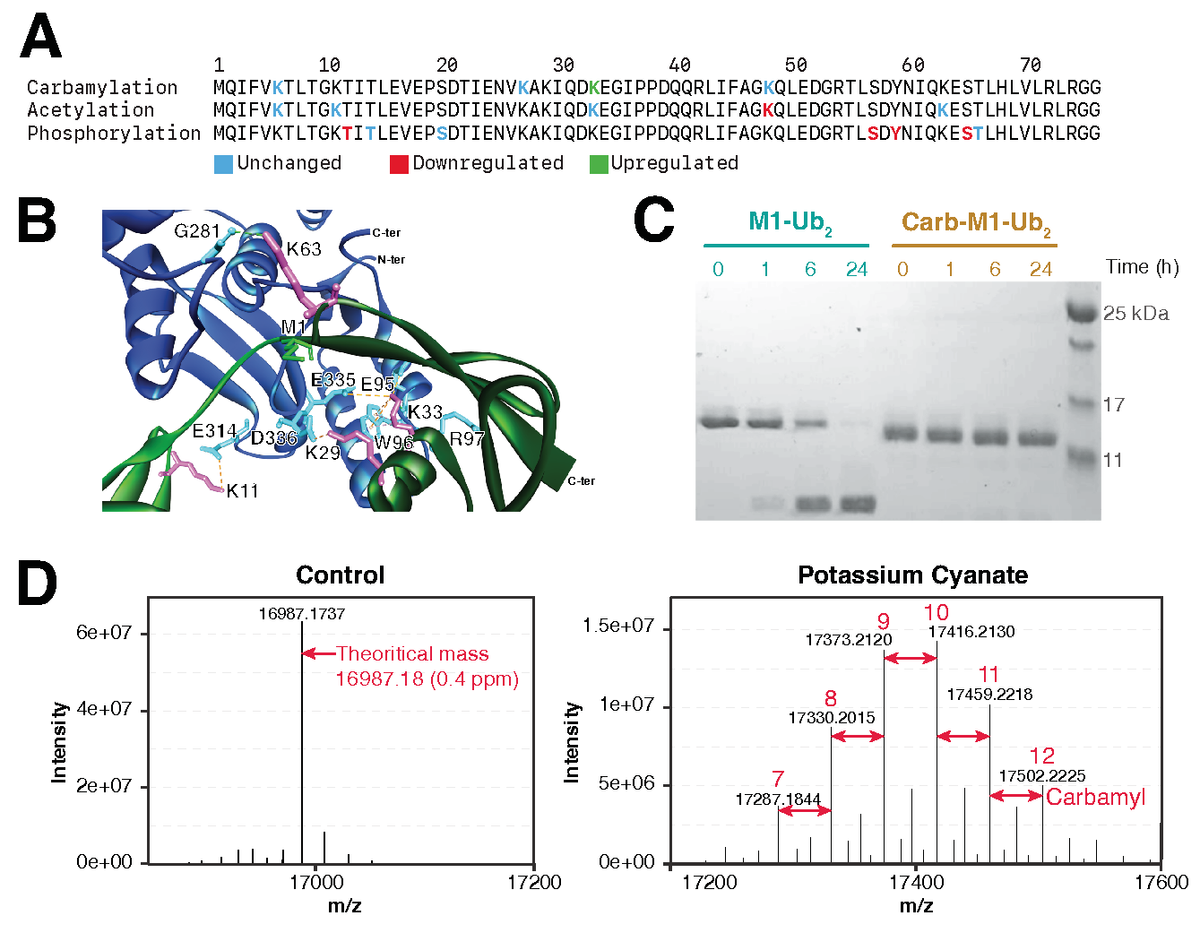
Ubiquitination carbamylation, acetylation, and phosphorylation sites, their regulation by bacterial lipopolysaccharide, and the effect of carbamylation in deubiquitination. (a) Carbamylated, acetylated, or phosphorylated residues on ubiquitin and their regulation by the LPS treatment. (b) Interactions between lysine residues of M1-linear diubiquitin and OTULIN. Proximal ubiquitin and distal ubiquitin are shown in dark and light green ribbon structures, respectively. OTULIN ribbon structure is shown in blue. Lysine residues of M1-linear diubiquitin and residues of OTULIN in magenta and cyan, respectively. Met-1 of the distal ubiquitin, which is linked to the proximal ubiquitin C-terminus, is highlighted as green line structure. Hydrogen bonds, electrostatic interactions, and hydrophobic interactions are shown in green, orange, and pink dashed lines, respectively. All the interactions were identified using Discovery Studio Visualizer 4.5 program. (c) SDS-PAGE of unmodified and carbamylated M1-linear diubiquitin chains incubated with OTULIN. Image is representative of 3 replicates. (d) Mass spectra of unmodified and carbamylated M1-linear diubiquitin chains.

We next focused our attention on ubiquitin since this protein was heavily modified by carbamylation, acetylation, and phosphorylation, including sites of each PTM that were regulated by the LPS treatment (Fig. 6A). Because of the ubiquitin role in regulating inflammatory signaling, we asked if any of the carbamylation sites were on lysine residues that might interfere with interaction with other proteins. We examined the complex structure of linear M1-linear diubiquitin bound with the deubiquitinase OTULIN (PDB accession number 3ZNZ) since it is a mechanism of shutting off NF-κB inflammatory signaling [17]. K29, K33, K63 from proximal ubiquitin unit and K11 from the distal one interface with OTULIN within to 2.6 to 5.2 Å by forming hydrogen bonds, electrostatic interactions, or hydrophobic interactions (Fig. 6B). Of these sites, K33 had its carbamylation levels reduced by the LPS treatment (Fig. 6A). To determine if ubiquitin carbamylation can interfere in OTULIN activity, we carbamylated M1-linear diubiquitin with potassium cyanate and incubated with OTULIN for various times. Deubiquitinase activity was assessed by the increase in deconjugated ubiquitin units were analyzed via SDS-PAGE, which indicated that carbamylation completely abolished M1-linear diubiquitin cleavage by OTULIN (Fig. 6C). The carbamylation efficiency was assessed by mass spectrometry and showed the addition of 7–12 carbamyl groups to the M1-linear diubiquitin (Fig. 6D). These results show that carbamylation regulates protein ubiquitination, at least in *vitro* conditions.

## Discussion

Carbamylation has been viewed by the proteomics community as a major artifact and confounding factor for lysine acetylation analysis by leading to mis-identification and affinity co-purification with anti-acetyllysine antibodies (hence decreased specificity and overall effectiveness in acetylation analysis). Here, we showed that the affinity co-purification with anti-acetyllysine antibodies can be effectively used to study the endogenous carbamylome of a cell. With minor modifications in sample preparation protocol and data analysis, we showed that it is possible to identify and quantify over 7,000 acetylated peptides in addition to over 8,000 carbamylated peptides. This opens opportunity to understand the physiological roles of carbamylation *in vivo*.

The motif analysis showed an enrichment in glutamate, aspartate, and phenylalanine residues close to the carbamylation site. Since carbamylation is non-enzymatic, we believe that these residues can help attract the chemical donor to the modification site. For instance, carbamoyl-phosphate synthase, which produces the carbamylation donor carbamoyl-phosphate, has phenylalanine and glutamate in its catalytic pocket [18]. Myeloperoxidase, which produces the carbamylation donor isocyanic acid, has multiple aspartates and glutamates in its catalytic pocket [19]. We found that carbamylation motifs partially overlap with the acetylation ones. This is expected to some extent. Like carbamylation, acetylation can also occur non-enzymatically. Indeed, in bacteria the major mechanism of lysine acetylation occurs non-enzymatically by acylation of amine groups with acetyl-phosphate [20], a carbamoyl-phosphate analog that increases with the excess of carbon availability in cells. The presence of acetyl-phosphate has not been reported in mammalian cells. However, acetyl-coA, the universal donor for acetyltransferases, can also induce acetylation non-enzymatically, mainly in CoA-binding proteins [21]. Carbamoyl-phosphate is produced in cells as intermediates of arginine and nucleotide synthesis metabolism. Levels of carbamoyl-phosphate increase in cells with excess of nitrogen availability to increase the production of arginine, which is used as an intermediate for urea production and subsequent secretion in urine [22]. Therefore, acetylation and carbamylation could represent an alternating mechanism of protein regulation in carbon and nitrogen excess, respectively.

Our data also showed an extensive co-modification of proteins with carbamylation, acetylation and phosphorylation. We found that the ubiquitination machinery to be one of those pathways that were co-modified and regulated by the LPS treatment. Different modifications have been shown to occur in ubiquitin. For instance, phosphorylation of Thr-12 on ubiquitin unit modifying histone H2A has been shown to regulate DNA damage response [23]. Moreover, phosphorylation of Ser-65 inhibits polyubiquitin formation and deconjugation of K63-linked polyubiquitin chains by deubiquitinases [24]. Lysine acetylation inhibits polyubiquitin chain elongation. This phenomenon is not only due to blocking the modification site since it also inhibits ubiquitination of other lysine residues [25]. It has been recently reported that ubiquitin carbamylation inhibits polyubiquitin formation [26]. We now show that protein carbamylation blocks M1-linear ubiquitin chains to be deconjugated by the deubiquitinase OTULIN. The downregulation of K33 carbamylation 24 h after LPS treatment may play a role in regulating inflammation by increasing the activity of OTULIN. However, whether all these modifications work together or in specific processes during inflammation still needs to be further studied.

In conclusion, we developed a method to analyze the carbamylomes of samples within a pipeline that simultaneously analyzes acetylomes and phosphoproteomes. This opens opportunities to study post-translational modification crosstalk and novel functions of carbamylation in cells.

## Data Availability

Mass spectrometry raw data was deposited into the MassIVE repository, which is a member of the ProteomeXchange Consortium. MassIVE accession: MSV000092020 Server: massive.ucsd.edu User: MSV000092020 Password: Acetyl4872

## Abbreviations

IMAC: immobilized metal affinity chromatography
LC-MS/MS: liquid chromatography tandem-mass spectrometry
LPS: lipopolysaccharide
PTM: post-translational modification
SDC: sodium deoxycholate
TMT: tandem mass tag
